# Efficacy and safety of local glucocorticoids for the treatment of acute radiation-induced intestinal injury: protocol of a multicenter randomized controlled trial

**DOI:** 10.3389/fphar.2025.1529977

**Published:** 2025-05-30

**Authors:** Qianqian Li, Xiaofeng Liu, Lichun Shao, Xiaohua Bao, Cheng Bai, Junchen Ge, Dongshuai Su, Wenda Xu, Ran Jiang, Zhenbin Mu, Hong Dai, Bingguo Piao, Yufeng Zhang, Haijun Liang, Ying Yan, Cheng Zhang, Xiangbo Xu, Jiang Chen, Jing Wang, Jian Zhang, Jie Zhang, Yibo Zhang, Jing Wang, Qingyu Yang, Xiaoqin Li, Xiaoyu Zhang, Yumei Hou, Ruosi Jiang, Huafeng Jin, Xingshun Qi, Huishan Wang

**Affiliations:** ^1^ Department of Gastroenterology, General Hospital of Northern Theater Command, Shenyang, China; ^2^ Department of Gastroenterology, The 960th Hospital of the Chinese People’s Liberation Army, Jinan, China; ^3^ Department of Gastroenterology, Air Force Hospital of Northern Theater Command, Shenyang, China; ^4^ Department of Gastroenterology, The 964th Hospital of the Chinese People’s Liberation Army, Changchun, China; ^5^ Department of Gastroenterology, The 967th Hospital of the Chinese People’s Liberation Army, Dalian, China; ^6^ Department of Gastroenterology, The 962nd Hospital of the Chinese People’s Liberation Army, Harbin, China; ^7^ Respiratory and Digestive Department, The 963rd Hospital of the Chinese People’s Liberation Army, Jiamusi, China; ^8^ Department of Gastroenterology, The 81th Group Military Hospital of of the Chinese People’s Liberation Army, Zhangjiakou, China; ^9^ Department of Gastroenterology, The 966th Hospital of the Chinese People’s Liberation Army, Dandong, China; ^10^ Department of Gastroenterology, The 92493 Hospital of the Chinese People’s Liberation Army, Huludao, China; ^11^ Department of Internal Medicine, Liaoning Provincial Corps Hospital of the Chinese People’s Armed Police, Shenyang, China; ^12^ Department of Gastroenterology, The 78th Group Military Hospital of the Chinese People’s Liberation Army, Mudanjiang, China; ^13^ Department of Gastroenterology, The 79th Group Military Hospital of of the Chinese People’s Liberation Army, Liaoyang, China; ^14^ Endoscopy Center, The 96605 Hospital of the Chinese People’s Liberation Army, Tonghua, China; ^15^ Department of Radiation Oncology, General Hospital of Northern Theater Command, Shenyang, China; ^16^ Department of General Surgery, General Hospital of Northern Theater Command, Shenyang, China; ^17^ Department of Cardiovascular Surgery, General Hospital of Northern Theater Command, Shenyang, China

**Keywords:** glucocorticoids, enema, intestinal injury, radiotherapy, randomized controlled trial

## Abstract

**Background and aims:**

Acute radiation-induced intestinal injury (ARII) usually occurs after pelvic radiotherapy. Glucocorticoids are potentially effective for the treatment of ARII due to their anti-inflammatory properties, but the currently available evidence is insufficient. Herein, we have designed a multicenter randomized controlled trial (RCT) to explore the efficacy and safety of glucocorticoids for the treatment of ARII.

**Methods:**

Overall, 60 patients with ARII will be enrolled. Eligible patients will be stratified according to the grade of ARII and randomly assigned at a ratio of 1:1 to the routine treatment alone and in combination with local glucocorticoids groups. The primary endpoints include the recovery and improvement of ARII. The secondary endpoints include the recurrence and aggravation of ARII and adverse events of glucocorticoids.

**Discussion:**

This RCT will provide high-quality evidence to clarify the role of local glucocorticoids for the treatment of ARII.

**Clinical trial registration:**

ClinicalTrials.gov, identifier NCT06410443.

## 1 Introduction

Radiotherapy, one of the mainstay treatments for malignant solid tumors in clinical practice, is necessary in nearly half of patients who receive anti-tumor therapy (Citrin, 2017). In spite of constant improvement in radiotherapy-related technology, toxic side effects of radiation on normal tissues are still unavoidable. Considering intestine as a radiation-sensitive organ, radiation-induced intestinal injury (RII) is frequently observed in patients who receive pelvic radiotherapy (Hauer-Jensen et al., 2014). Acute RII (ARII) often refers to significantly progressive abdominal pain, diarrhea, and tenesmus with 3 months since the course of radiotherapy (Hauer-Jensen et al., 2014; Abu-Sbeih et al., 2023; Hovdenak et al., 2000). Serious complications, such as perforation, obstruction, and fistula, may also develop, compromising the patients’ quality of life and outcomes (Gami et al., 2003).

ARII is pathologically characterized as cell death and inflammation response in acute phase. Radiation can damage not only tumor cells by directly or indirectly affecting the synthesis of DNA chains, but also intestinal epithelial cells due to their relatively high mitotic capacity (Spyropoulos et al., 2011). Death of epithelial cells leads to intestinal mucosal breakdown and further induces inflammation. Infiltration of inflammatory cells can be observed in the lamina propria (Hovdenak et al., 2000; Starzewski et al., 2006). Inflammatory process is then amplified by the recruitment and transmigration of monocytes and the activation of resident mast cells, stimulating the production of pro-inflammatory cytokines, such as interleukin (IL) and tumor necrosis factor-α (TNF-α) (Francois et al., 2013).

According to the current practice guidelines, glucocorticoids have been widely recommended for the management of inflammatory bowel disease due to their anti-inflammatory effects (Lamb et al., 2019). It seems that glucocorticoids should also be potentially effective for ARII, considering the importance of inflammation response in the development and progression of ARII. However, the existing evidence on the use of glucocorticoids in such patients is still inconsistent among studies (Chen et al., 2021; Kochhar et al., 1991). Besides, the current practice guideline and consensus mostly focus on the management of chronic radiation-induced intestinal injury (CRII), rather than ARII (Paquette et al., 2018; Wang and Wang, 2018). Thus, we aim to conduct a multicenter randomized controlled trial (RCT) to explore the efficacy and safety of glucocorticoids for the treatment of ARII.

## 2 Methods

### 2.1 Study design

**FIGURE 1:**
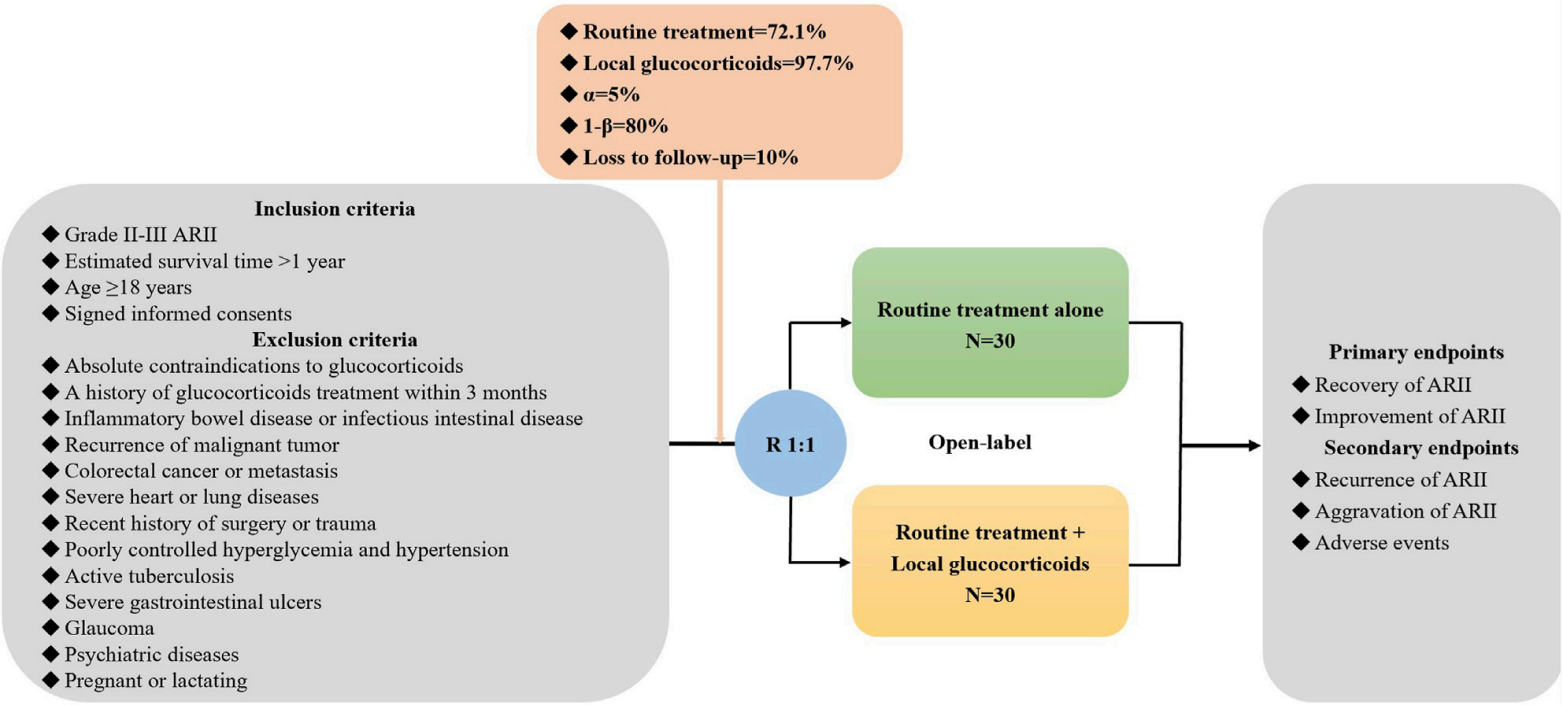
Flowchart of this trial.

In this multicenter RCT, patients with grade II-III ARII will be screened. The investigators from each center will sufficiently explain the objectives and harms of this study to potentially eligible participants. Once the participants agree and sign written informed consents, they will be randomly assigned at a ratio of 1:1 to routine treatment alone and in combination with local glucocorticoids groups. The data will be collected at admission, after treatment, and during follow-up periods (Figure 1). This study follows the Declaration of Helsinki, and the ethical approval number from medical ethical committee of the General Hospital of Northern Theater Command is Y (2024) 052-1. It has been registered in the *ClinicalTrials.gov.* Website (registration number: NCT06410443).

### 2.2 Eligibility criteria

The inclusion criteria include: (1) grade II-III ARII; (2) estimated survival time >1 year; (3) age ≥18 years; and (4) signed informed consents.

The exclusion criteria include: (1) absolute contraindications to glucocorticoids; (2) a history of glucocorticoids treatment within 3 months; (3) inflammatory bowel disease or infectious intestinal disease; (4) recurrence of malignant tumor; (5) colorectal cancer or metastasis; (6) severe heart or lung diseases; (7) recent history of surgery or trauma; (8) poorly controlled hyperglycemia and hypertension; (9) active tuberculosis; (10) severe gastrointestinal ulcers; (11) glaucoma; (12) psychiatric diseases; and (13) pregnant or lactating.

### 2.3 Randomization, assignment, and blinding

Participants are stratified according to the grade of ARII at admission, and then randomly assigned to routine treatment alone and in combination with local glucocorticoids groups at a ratio of 1:1 within each stratum. The randomization sequence will be generated by computer system. The trial-group assignments are open-label to the participants and physicians.

### 2.4 Treatment

Routine treatment includes antibiotics, probiotics, vitamins, antidiarrheal drugs, enteral nutrition, and endoscopic argon plasma coagulation according to the current practice guideline (Wang and Wang, 2018). These drugs and procedures are selected based on the patients’ disease conditions and their availability at each site.

Local glucocorticoids refer to retention enema of dexamethasone 10 mg combined with 100 mL of 0.9% sodium chloride injection once daily for a duration of 7 days. It requires the solution to be retained in the bowel for more than 1 h.

### 2.5 Study endpoints

The primary endpoints include the recovery and improvement of ARII after treatment. The secondary endpoints include the recurrence and aggravation of ARII, and adverse events during hospitalization and follow-up periods.

### 2.6 Sample size calculation

Sample size is calculated by using significant difference test in the proportion between two groups on the statistical software PASS 15.0.5 (NCSS, LLC. Kaysville, Utah, United States). The rates of improvement of ARII are estimated as 97.7% and 72.1% in patients treated with and without glucocorticoids according to the results of a previous study by Chen et al. (2021) An alpha value of 0.05, a power of 80%, and a dropout rate of 10% are set. Finally, 30 patients will be required for each group.

### 2.7 Diagnosis, grade, and definitions

The diagnosis of ARII is mainly based on the comprehensive analysis of clinical, endoscopic, imaging, and histopathological manifestations after excluding infectious and other non-infectious enteritis. Notably, a history of radiotherapy for pelvic malignant tumors is a required diagnostic criterion.

The grade of ARII refers to the radiation injury classification proposed by the Radiation Therapy Oncology Group (RTOG) and the European Oncology Radiation Therapy Group (EORTG) (Cox et al., 1995), as follows.

(1) Grade 0 is defined as no abdominal symptom.
(2) Grade I is defined as increased frequency of defecation, loose stools, and/or rectal discomfort not requiring medication.
(3) Grade II is defined as diarrhea requiring parasympatholytic drugs; and/or rectal secretion not requiring sanitary pads; and/or abdominal or rectal pain requiring analgesic drugs.
(4) Grade III is defined as diarrhea requiring parenteral nutrition support; and/or secretion or hematochezia requiring sanitary pads; and/or abdominal distension showing dilatation of intestinal loop on abdominal X-rays.
(5) Grade IV is defined as acute or subacute intestinal obstruction, fistula, or perforation; and/or hematochezia requiring blood transfusion; and/or abdominal pain requiring gastrointestinal decompression or surgical intervention.

The grade of intestinal mucosal damage on colonoscopy is evaluated in accordance with the Vienna rectal score (Wachter et al., 2000), as follows.

(1) Grade 0 is defined as only localized mucosal hyperemia and edema.
(2) Grade 1 is defined as diffuse non-confluent mucosal hyperemia and edema with single telangiectasia but without ulcer, stenosis, and necrosis.
(3) Grade 2 is defined as diffuse and confluent mucosal hyperemia and edema with multiple non-confluent telangiectasia but without ulcer, stenosis, and necrosis.
(4) Grade 3 is defined as unlimited mucosal hyperemia and edema with multiple and confluent telangiectasia, and the area of superficial ulcer is less than 1 cm^2^, but without stenosis and necrosis.
(5) Grade 4 is defined as unlimited mucosal hyperemia, edema, and telangiectasia, and the area of superficial ulcer is more than 1 cm^2^, and the diameter of lumen diameter is more than 2/3 of the regular lumen diameter, but without necrosis.
(6) Grade 5 is defined as unlimited mucosal hyperemia, edema, and telangiectasia with deep ulcer, fistula, or perforation, and the diameter of lumen diameter is less than 1/3 of the regular lumen diameter, and presence of necrosis.

Recovery is defined as patients’ clinical symptoms and intestinal mucosal damage on colonoscopy disappear. Improvement is defined as patients’ clinical symptoms alleviate or the Vienna rectal score decreases at least one grade.

### 2.8 Data collection

The following data will be collected: (1) demographic data (i.e., gender and age); (2) history of malignancy; (3) interval between radiotherapy and occurrence of ARII; (4) history and type of medications for ARII before admission; (5) height, weight, and vital signs (i.e., body temperature, heart rate, blood pressure, and respiratory rate); (6) clinical symptoms (i.e., abdominal pain, distension, diarrhea, frequency of defecation, tenesmus, hematochezia, mucous stool, nausea, vomiting, acid reflux, and heartburn); (7) laboratory data (i.e., red blood cells, hemoglobin, white blood cells, neutrophils, lymphocytes, procalcitonin, c-reactive protein, IL-6, IL-2, TNF-α, IFN-γ, potassium, sodium, calcium, iron, and stool routine test); (8) grade of intestinal mucosal damage on colonoscopy; (9) histology of colorectal mucosa; and (10) intestinal obstruction, fistula, perforation, and/or stricture.

### 2.9 Follow-up

All participants will be followed on the 1st, 3rd, 6th, and 12th month after treatment. Clinical symptoms, physical examinations, and laboratory tests will be collected. Adverse events will be monitored. Survival status will be recorded, including the major cause and date of death.

### 2.10 Treatment termination

Causes of treatment termination include: (1) participants request to terminate their assigned treatment; (2) participants’ conditions worsen and are not appropriate to continue their assigned treatments; (3) participants’ adherence is poor; (4) adverse events occur; and (5) pregnancy. In this setting, the assigned treatment will be discontinued, but the follow-up observation of these participants is still conducted.

### 2.11 Withdrawal

Participants can withdraw from the trial at any time due to the following reasons: (1) participants withdraw their informed consents; (2) study termination or center closure; (3) loss to follow up; and (4) death.

### 2.12 Study schedule

The study treatment and assessment plan are summarized in Table 1.

(1) Eligible participants will be enrolled after signing their informed consents.
(2) Demographic data, medical history, clinical symptoms, physical examinations, laboratory tests, abdominal computed tomography (CT) images, and description under colonoscopy will be collected before treatment.
(3) Repeated abdominal CT scans will be performed on the 6th month after treatment.
(4) Repeated colonoscopy will be performed on the 3rd month and 12th month after treatment.
(5) Adverse events and survival status will be collected on the 7th day, 1st month, 3rd month, 6th month, and 12th month after treatment.

**TABLE 1:** Study schedule.

Procedure	Screening	Treatment period 7th day	Follow-up period			
			1st month	3rd month	6th month	12th month
Informed consent	√					
Eligibility criteria	√					
Demographic data	√					
Medical history	√					
Clinical symptoms	√	√	√	√	√	√
Physical examinations	√	√	√	√	√	√
Laboratory tests	√	√	√	√	√	√
Abdominal computed tomography	√				√	
Colonoscopy	√			√		√
Adverse events		√	√	√	√	√

### 2.13 Adverse events

Investigators should determine whether adverse events are related to glucocorticoids. Adverse events related to glucocorticoids mainly include infection, water-sodium retention, Cushing syndrome, poorly controlled hyperglycemia, gastrointestinal ulcer, thromboembolic disease, neuropsychiatric symptoms, osteoporosis, increased intraocular pressure, and withdrawal syndrome. If an adverse event occurs, the investigator should give appropriate medical interventions, including symptomatic treatment, dose reduction or discontinuation of glucocorticoids, and follow-up observations until the adverse event will be completely resolved. Information related to adverse events will be recorded in detail, including start and end dates, symptoms and signs, severity and frequency, management, and outcomes.

### 2.14 Protocol deviations and violations

If a procedure or behavior was not in accordance with the protocol, but did not cause any significant impact on the study endpoints, it would be defined as a minor protocol deviation, such as missing a visit window which does not affect the use of assigned treatment and assessment of efficacy per the protocol. If a procedure or behavior was not in accordance with the protocol, and led to an impact on the study endpoints or participants’ safety, it would be defined as a major protocol violation, such as inadequate informed consent, unmet eligibility criteria, unreported serious adverse events, use of prohibited medication, or missing of multiple visits.

### 2.15 Data analysis

The intention-to-treat (ITT) set comprises all participants randomized. The per-protocol (PP) set consists of participants who complete the assigned treatments of protocol and develop the primary endpoint events without major protocol violation. The safety set contains participants who receive at least one dose of local glucocorticoids and undergo at least one safety evaluation. Continuous variables are described as mean ± standard deviation and median (range) and categorical variables are described as frequencies (percentages). Whitney U test, chi-square test, and Fisher’s exact test are used, if appropriate. A two-sided P value of <0.05 is considered statistically significant. All statistical analyses are performed using IBM SPSS 26.0 software (IBM Corp, Armonk, NY, United States).

## 3 Discussion

Glucocorticoids can inhibit the inflammatory response by balancing pro-inflammatory and anti-inflammatory cytokines (De Iudicibus et al., 2011), which may partly explain their beneficial effects on severe RII. Successful cases have been reported for decades, and the potential role of glucocorticoids for severe RII has also been suggested in comparative studies (Loiudice and Lang, 1983; Macdonald and Hoyt, 1956). The Chinese guideline strongly recommends glucocorticoids for the treatment of hemorrhagic radiation rectal injury (Wang and Wang, 2018). However, the paper cited for this guideline recommendation actually explored the efficacy of prednisolone combined with sulfasalazine versus sucralfate for hemorrhagic radiation rectal injury (Kochhar et al., 1991). More importantly, the paper cited concluded that sucralfate was a more suitable therapeutic option. By comparison, the American clinical practice guideline did not recommend glucocorticoids as a medical option due to the absence of high-quality evidence (Paquette et al., 2018). Beyond that, available evidence on glucocorticoids is primarily obtained from patients with CRII.

Most of patients with ARII present with mild symptoms and do not require medical care, but 5%–15% of them have to terminate radiotherapy due to severe discomfort, and even develop complications (Hauer-Jensen et al., 2004). In a previous RCT, Chen et al. demonstrated anti-inflammatory effects of glucocorticoids on ARII (Chen et al., 2021). Sixty-eight patients with ARII were randomly assigned to receive either dexamethasone combined with montmorillonite retention enema or oral montmorillonite. Inflammatory cytokines (IL-2 and IFN-γ) levels significantly decreased after treatment, with a more pronounced advantage observed in patients receiving combination dexamethasone (P < 0.001). They also demonstrated more significant response to treatment in terms of clinical improvement (97.67% vs. 72.09%, P < 0.001) and mucosal repairment under colonoscopy (93.02% vs. 65.12%, P < 0.001). Therefore, Chen et al. concluded that patients with ARII could receive more benefits from dexamethasone combined with montmorillonite. However, the evaluation of mucosal repairment, an important criterion for efficacy, was subjective, and lacked detailed descriptions. By contrast, another RCT by Kochhar et al. found a better efficacy of sucralfate than glucocorticoids (Kochhar et al., 1991). Thirty-seven patients were randomly assigned to receive either prednisolone enema combined with oral sulfasalazine or sucralfate enema. Although both two regimens were effective for alleviating clinical symptoms, prednisolone combination treatment was inferior to sucralfate (53.3% vs. 94.1%, P < 0.05). In terms of endoscopic improvement, there was no significant difference between the two groups (46.7% vs. 70.5%, P > 0.05). But two patients withdrew from the trial due to intolerable myalgia and headache in prednisolone enema combined with oral sulfasalazine treatment group. Considering the safety and low cost of sucralfate, Kochhar et al. preferred sucralfate as the better choice for RII (Kochhar et al., 1991). Notably, some of the patients included in the study by Kochhar et al. had CRII with intestinal tissue fibrosis, where anti-inflammatory effects of glucocorticoids might be unsatisfactory.

Patients with malignant tumors may be at poor physical conditions. Although glucocorticoids have been used for the management of tumor-related pain and edema and side effects caused by chemotherapy, such as nausea and allergies (Kalfeist et al., 2022), its safety profile should be recognized. Adverse events of glucocorticoids have not been discussed yet in patients with ARII.

In conclusion, this study is promising to provide new evidence to explore the efficacy and safety of glucocorticoids for the treatment of ARII.

## Abbreviations

RII, Radiation-induced intestinal injury; ARII, Acute radiation-induced intestinal injury; IL, Interleukin; TNF-α, Tumor necrosis factor-α; CRII, Chronic radiation-induced intestinal injury; RCT, Randomized controlled trial; RTOG, Radiation Therapy Oncology Group; EORTG, European Oncology Radiation Therapy Group; CT, Computed tomography; ITT, Intention-to-treat: PP, Per-protocol.
